# Overall survival of cancer patients with serum lactate dehydrogenase greater than 1000 IU/L

**DOI:** 10.1007/s13277-016-5228-2

**Published:** 2016-08-10

**Authors:** Rujiao Liu, Jun Cao, Xiang Gao, Jian Zhang, Leiping Wang, Biyun Wang, Lin Guo, Xichun Hu, Zhonghua Wang

**Affiliations:** 1 Department of Medical Oncology, Fudan University Shanghai Cancer Center, Shanghai, China; 2 Department of Oncology, Shanghai Medical College, Fudan University, 270 Dong’An Road, Shanghai, 200032 China; 3 Department of Clinical Laboratory, Fudan University Shanghai Cancer Center, Shanghai, China; 4 Shanghai Medical College, Fudan University, Shanghai, 200032 China

**Keywords:** Lactate dehydrogenase, Metastatic cancer, Prognostic factor, Overall survival, Palliative care

## Abstract

High level of serum lactate dehydrogenase (LDH) is a well-known poor prognostic factor in patients with malignancies. However, there was no data on overall survival (OS) in cancer patients with serum LDH level > 1000 IU/L, and the prognostic value of the changes in LDH over time for OS had not been reported. Clinical data of 311 cancer patients with metastatic disease with serum LDH >1000 IU/L (four times upper limit of normal) admitted consecutively to a single center were reviewed in this retrospective study. LDH level ranged from 1002 to 8235 U/L with a mean of 1689 U/L. The median OS was 1.7 months (95 % CI: 1.4–2.0). About half of patients (*n* = 163, 52 %) died within 2 months with the median OS of 0.5 months (95 % CI: 0.3–0.7). Only 173 patients were indicated for salvage treatment. Fifty-one patients’ serum LDH level decreased to normal at 2 months following chemotherapy; OS was significantly longer in these patients (22.6 months, 95 % CI: 10.9–34.3, *p* < 0.001) compared to those with persistently abnormal serum LDH at 2 months (4.0 months, 95 % CI: 3.4–4.6). The independent factors that increased the death risk were ECOG performance status 3–4 (HR: 2.05, 95 % CI: 1.42–2.97, *p* < 0.001), supportive care only (HR: 2.91, 95 % CI: 2.06–4.10, *p* < 0.001), and persistently abnormal serum LDH at 2 months (HR: 2.72, 95 % CI: 1.67–4.42, *p* < 0.001). In conclusion, serum LDH level > 1000 IU/L predicted a terminal stage in metastatic cancer patients. OS was significantly prolonged in patients indicated for effective palliative treatment and LDH level decreased to normal at 2 months.

## Introduction

Lactate dehydrogenase (LDH) is a metabolic enzyme widely expressed in different tissues and is detectable in serum, which catalyzes the interconversion of pyruvate and lactate during glycolysis and gluconeogenesis [1]. It has long been known that many human cancers have higher LDH levels than normal tissues [1]. Increased LDH may also be a prognostic tumor marker in many other solid tumors, including colorectal cancer [2], nasopharyngeal carcinoma [3, 4], lung cancer [5–7], breast cancer [8, 9], prostate cancer [10], germ cell cancer [11, 12], and melanoma [13, 14]. The serum level of LDH correlated with tumor burden and was thought to reflect the tumor’s growth and invasive potential [15]. It has long been appreciated that LDH is a prognostic factor for survival, being one of the five clinical features known as the international prognostic index (IPI) for non-Hodgkin’s lymphoma [16] and the Memorial Sloan-Kettering Cancer Center (MSKCC) risk classification for metastatic renal cell carcinoma [17].

The determination of remaining life expectancy is of great importance for patients with advanced cancer, both in guidance of cancer therapies and providing information for patients and their relatives. Regardless of primary cancer type, patients with advanced cancer have much in common, such as fatigue, pain, and heavy tumor burden. Few studies have evaluated the value of LDH >1000 IU/L (four times upper limit of normal, ULN) as a predictor of survival time in metastatic cancer patients.

In this retrospective study, overall survival (OS) of cancer patients with serum LDH greater than 1000 IU/L at baseline was evaluated. Correlation between better clinical outcomes and decrease of baseline serum LDH to normal level after 2 months was also evaluated after adjustment with other clinical features.

## Patients and methods

### Study population

Medical records of all cancer patients with histologically confirmed primary and/or metastatic disease (including solid tumors and lymphoma) admitted to the Fudan University Shanghai Cancer Center (Shanghai, China) consecutively from January 2005 to May 2015 were reviewed. All patients whose serum LDH level detected for the first time to be greater than 1000 IU/L (4 × ULN) were included into this study. The exclusion criteria included: incomplete medical history or suffering from two or multiple primary malignant neoplasm. Clinical features such as age, ECOG performance status, treatment (chemotherapy or palliative care), and serum LDH (baseline and post 2-month) were collected. Patients were categorized into three groups based on serum LDH level at 2 months after baseline: decrease to normal, persistently abnormal, and died within 2 months. Written informed consent was obtained from all patients. The study was approved by the Institutional Review Board of the Fudan University Shanghai Cancer Center.

### Laboratory measurement of serum LDH

LDH measurement was performed locally at our cancer center using Roche cobas 8000. The normal range is 120–250 U/L. Once the patients were enrolled, serum LDH was collected at least every 4 weeks. Serum LDH was detected with the same machine from 2005 to 2015, and with the same normal range.

### Statistical analysis

The primary objectives of this analysis were to determine the OS for patients with metastatic disease whose LDH >1000 IU/L and to evaluate whether changes in LDH over time were a potential prognostic and a predictive biomarker for OS. OS was defined as the interval between the day when LDH level > 1000 IU/L was detected at the first time during admission to this hospital and the date of death or last follow-up visit if the patients were still alive.

Mean serum level of LDH for each subgroup was compared by standard analysis-of-variance (ANOVA) procedures. Survival curves were estimated by the Kaplan-Meier method and analyzed by the log-rank test. Cox regression was used for univariate and multivariate survival analyses, and a reduced model was applied using stepwise backward elimination until only significant (*P* < 0.05) variables remained in multivariate survival analysis. A *p* value of <0.05 was considered statistically significant. Statistical analyses were performed using the Statistical Package for the Social Sciences version 12.0.

## Results

### Patients

A total of 311 patients with serum LDH >1000 IU/L were enrolled into this study. Patients’ characteristics (as baseline) were shown in Table 1. The majority of patients (*n* = 198, 64 %) had ECOG scoring of 3, and seven patients (2 %) had 4. The most commonly seen tumor of origin was gastrointestinal cancer (34 %), followed by lymphoma (*n* = 57) (18 %), breast cancer (16 %), lung cancer (14 %), nasopharyngeal cancer (5 %), and others (13 %). The majority of patients (*n* = 254, 82 %) had metastatic solid tumors, except for 57 patients with lymphoma of which 27 patients were diagnosed with stage II–III. There was a significant difference between the three LDH groups in the ECOG performance status (*p* < 0.001), as well as the administration of treatment (*p* < 0.001) (Table 2).

**Table 1:** Patients’ characteristics at baseline (*n* = 311)

Features		Patients, n (%)	Baseline LDH level (U/L)		*p* value
			Mean	Range	
Age (years)	<65	258 (83 %)	1706	1002–8235	0.591
	≥65	53 (17 %)	1608	1010–6479	
Gender	Male	174 (56 %)	1713	1004–8235	0.613
	Female	137 (44 %)	1659	1002–5953	
ECOG	0–2	106 (34 %)	1549	1002–5006	0.058
	3–4	205 (66 %)	1758	1002–8235	
Treatment	Chemotherapy	173 (56 %)	1580	1002–5006	0.052
	Supportive care	138 (44 %)	1826	1002–8235	
Primary tumor	Gastrointestinal cancer	106 (34 %)	1714	1006–7817	0.083
	Lymphoma	57 (18 %)	1953	1002–8235	
	Breast cancer	50 (16 %)	1731	1002–5953	
	Lung cancer	43 (14 %)	1541	1004–2803	
	Nasopharyngeal cancer	15 (5 %)	1434	1019–2328	
	Others	40 (13 %)	1453	1017–3000

**Table 2:** Patients’ characteristics according to the serum LDH level

Features		Decrease to normal	Persistently abnormal	Died within 2 months	*p* value
Age (years)	<65	44 (86.3 %)	85 (87.6 %)	129 (79.1 %)	0.168
	≥65	7 (13.7 %)	12 (12.4 %)	34 (20.9 %)	
Gender	Male	27 (52.9 %)	54 (55.7 %)	93 (57.1 %)	0.873
	Female	24 (47.1 %)	43 (44.3 %)	70 (42.9 %)	
ECOG	0–2	42 (82.4 %)	57 (58.8 %)	7 (4.3 %)	<*0.001*
	3–4	9 (17.6 %)	40 (41.2 %)	156 (95.7 %)	
Treatment	Chemotherapy	51 (100 %)	82 (84.5 %)	40 (24.5 %)	<*0.001*
	Supportive care	0(0.0 %)	15 (15.5 %)	123 (75.5 %)	
Primary tumor	Gastrointestinal cancer	11 (21.6 %)	34 (35.1 %)	61 (37.4 %)	0.145
	Lymphoma	18 (35.3 %)	13 (13.4 %)	26 (16.0 %)	
	Breast cancer	8 (15.7 %)	18 (18.6 %)	24 (14.7 %)	
	Lung cancer	7 (13.7 %)	14 (14.4 %)	22 (13.5 %)	
	Nasopharyngeal cancer	1 (2.0 %)	6 (6.2 %)	8 (4.9 %)	
	Others	6 (11.8 %)	12 (12.4 %)	22 (13.5 %)	

There was a significant difference between the three LDH groups in the ECOG performance status (*p* < 0.001), as well as administration of treatment (*p* < 0.001)

### LDH changes over time and outcome

**Fig. 1:**
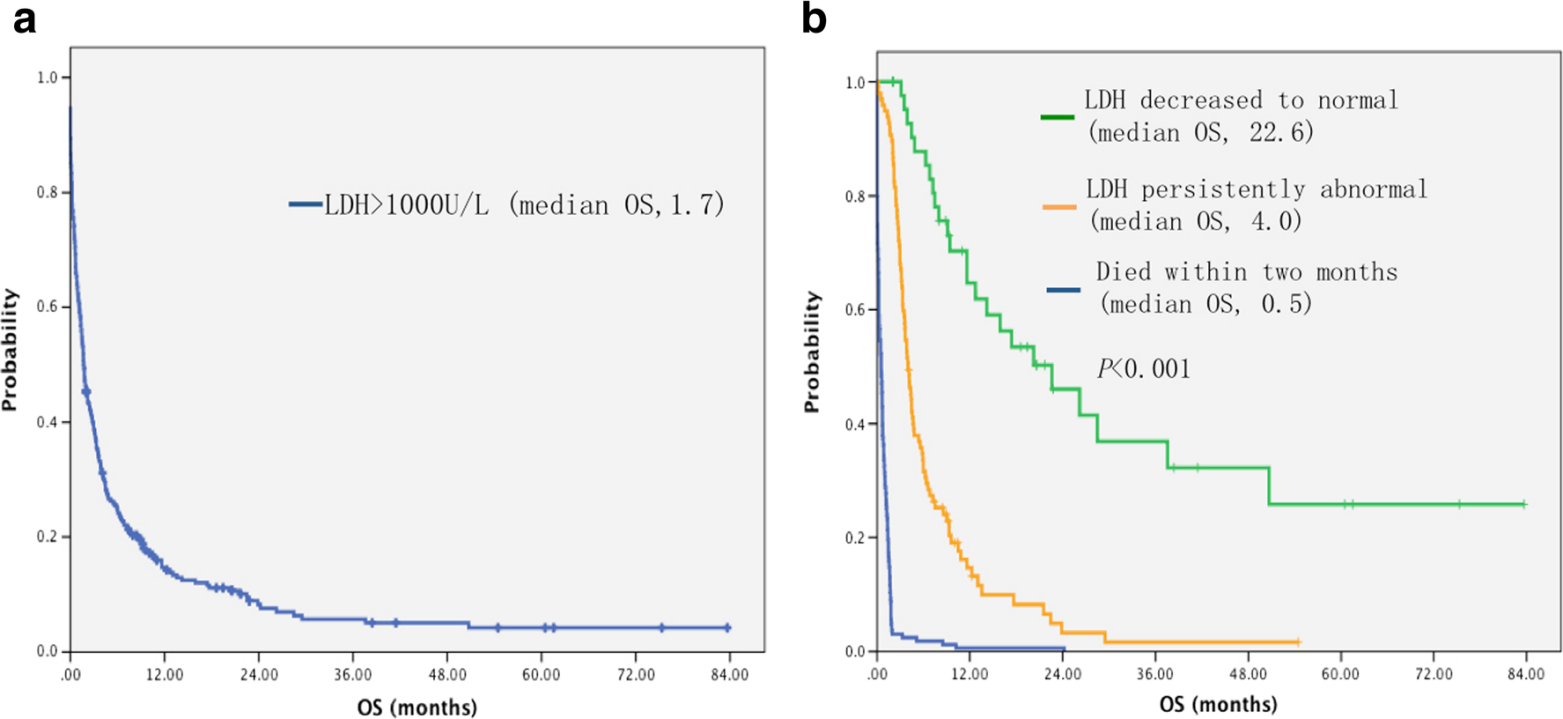
**a** Kaplan-Meier survival curves of all patients with baseline LDH >1000 IU/L. The median OS for patients with baseline LDH >1000 IU/L was only 1.7 months (95 % CI: 1.4–2.0). **b** Kaplan-Meier survival curves by patients’ LDH level at 2 months after baseline. Median overall survival, LDH decreased to normal at 2 months group: 22.6 months (95 % CI: 10.9–34.3); LDH persistently abnormal group: 4.0 months (95 % CI: 3.4–4.6); and died within 2 months group: 0.5 months (95 % CI: 0.3–0.7), respectively

Serum LDH level at baseline ranged from 1002 to 8235 U/L with a mean of 1689 U/L. About half of the patients (173, 56 %) had anti-tumor therapy while the others received supportive care. LDH >1000 IU/L predicted poor survival with a median OS of only 1.7 months was observed (95 % CI: 1.4–2.0, Fig. 1A), with an overall cumulative 1-year survival of 15.6 %.

Among the 311 patients, 163 patients (52 %) died within 2 months with the median OS of only 0.5 months (95 % CI: 0.3–0.7). About half of patients (148, 48 %) lived longer than 2 months and had the information of LDH after 2-month treatment. All patients were categorized into three groups according to LDH level at 2 months after baseline: decreased to normal, persistently abnormal, and died within 2 months. The 1-year survival rate for the three LDH groups was 64.7 %, 14.7 %, and 0.7 %, respectively (*p* < 0.001). LDH returned to normal (≤250 IU/L) at 2 months was observed in 51 (16 %) patients after chemotherapy with an OS of 22.6 months (95 % CI: 10.9–34.3), which was significantly longer than those with persistently abnormal serum LDH level (4.0 months, 95 % CI: 3.4–4.6, *p* < 0.001, Fig. 1b).

No positive association between different primary tumor of origin and OS was observed. However, further analysis of patients with lymphoma showed different OS for patients at different disease stage. Ten patients of stage II disease had significantly longer OS (17.7 months, 95 % CI: 10.7–24.7, *p* < 0.001) compared with 17 patients with stage III disease (1.5 months, 95 % CI: 0.1–2.9) and 30 patients with stage IV disease (1.1 months, 95 % CI: 0.7–1.5).

### Univariate and multivariate models of overall survival

There were no significant differences in OS according to age, gender, and primary tumor site in univariate analyses. Three variables were found to be associated with a significant increased in the risk of death, including poor ECOG scoring of 3–4 (*p* < 0.001), supportive care without chemotherapy (*p* < 0.001), and persistently abnormal serum LDH level at 2 months after baseline (*p* < 0.001). (Table 3).

**Table 3:** Univariate and multivariate models for overall survival in patients with initial LDH level > 1000 IU/L

Factor		Univariate			Multivariate		
		HR	95 % CI	*p* value	HR	95 % CI	*p* value
Age ≥ 65 years old		1.38	0.99–1.92	0.056			
Gender: female		0.83	0.65–1.07	0.149			
ECOG: 3–4 vs. 0–2		5.46	4.05–7.35	**<** *0.001*	2.05	1.42–2.97	**<** *0.001*
Treatment: support care vs. chemotherapy		7.49	5.61–10.02	**<** *0.001*	2.91	2.06–4.10	**<** *0.001*
Primary tumor: gastrointestinal cancer		1.00					
Lymphoma		0.82	0.57–1.18	0.288			
Breast cancer		1.03	0.71–1.49	0.883			
Lung cancer		1.23	0.83–1.83	0.308			
Others		1.05	0.70–1.58	0.826			
Nasopharyngeal cancer		1.57	0.85–2.88	0.148			
LDH at 2 months after baseline	Decreased to normal	1.00			1.00		
	Persistently abnormal	3.70	2.31–5.91	**<** *0.001*	2.72	1.67–4.42	**<** *0.001*
	Died within 2 months	22.34	13.80–36.15	**<** *0.001*	12.59	7.39–21.43	**<** *0.001*

The independent factors that increased the risk of death were ECOG performance status 3-4(HR: 2.05, *p* <0.001), supportive care only (HR: 2.91, *p* <0.001), and persistently abnormal LDH level at two months (HR: 2.72, *p* <0.001)

These three covariates were also significantly associated with poor survival after adjustment for all other variables in the full multivariate model. The independent factors that increased the risk of death were ECOG performance status 3–4 (HR: 2.05, 95 % CI: 1.42–2.97, *p* < 0.001), supportive care only (HR: 2.91, 95 % CI: 2.06–4.10, *p* < 0.001), and persistently abnormal LDH level at 2 months, (HR: 2.72, 95 % CI: 1.67–4.42, *p* < 0.001). (Table 3).

## Discussion

In the present study, the median OS was only 1.7 months for patients with extremely high level LDH >1000 IU/L. OS was significantly longer up to 22.6 months in patients who had tolerated effective salvage treatment and LDH level decrease to normal at 2 months.

In our daily clinical practice, the majority of patients with advanced or metastatic disease could be detected to have extremely high serum level of LDH (greater than 4 × ULN), where the oncologists often predicted these patients with a poor prognosis. Estimation of remaining life expectancy of patients with metastatic disease is one of the greatest concerns [18]. But no exact survival data could be given during the communication between doctors and patients. This study revealed that remaining life expectancy was extremely short for metastatic cancer patients with LDH greater than 4 × ULN. Hence, for those patients who could tolerate toxicities of chemotherapy, effective and sufficient chemotherapy was urgently needed.

It was reported that high LDH level was associated with poor therapy response [19]. However, in the present study, among the 173 patients receiving chemotherapy, 51 had serum LDH level decrease to normal after 2 months, achieving a significantly enhanced survival. This is a strong evidence to support effective chemotherapy of full dose even in patients with high LDH level.

Notably, the prognostic role of serum LDH in oncology has long been recognized. LDH is a key enzyme in the process of energy production in cancer cells, it catalyzes the conversion of pyruvate to lactate in hypoxic conditions [4]. Since its function in anaerobic metabolism, cancer cells grow even after their rapid proliferation that leads to low-oxygen conditions in the tumor microenvironment [20]. Thus, LDH plays an important role in tumor progression and maintenance [21, 22]; inhibition of LDH inhibits tumor progression and has been considered for the therapeutic target of cancer energy metabolism [21]. LDH levels are increased in response to tissue injury or during disease states; LDH could be a marker of tumor burden for advanced cancer patients [23]. Higher LDH level was related with shorter survival in various types of cancer. In patients with non-small cell lung cancer (*N* = 2531), normal baseline serum LDH levels were significantly associated with better survival [5]. Similar reports were seen in colorectal cancer [2], nasopharyngeal carcinoma [3, 4], breast cancer [8, 9], prostate cancer [10], germ cell cancer [11, 12], and melanoma [13, 14].

Although LDH has five isoforms with different distributions, total serum LDH assessment being a routine part of clinical work is convenient and cheap. Furthermore, total serum LDH successfully predicted survival in previous studies [2, 10, 11, 16], without the necessity to detect LDH isoforms separately [8].

Our study enrolled patients with serum level of LDH >1000 IU/L only. However, several studies had analyzed the cut off value of serum LDH level which could be determined as a prognostic factor, ranging from 220 IU/L in metastatic renal cell carcinoma to 470 IU/L in metastatic pancreatic cancer [24, 25]. The cut off value in the present article was much higher for we aimed to study the overall survival in patients with advanced cancer and heavy tumor burden, and identified patients with LDH greater than 1000 IU/L as patients in advanced stage according to previous studies. Brown et al. conducted a multivariate analysis from 233 patients with metastatic breast cancer, and LDH was statistically significant with increased risk of death. The risk of death was increased almost 1.6-fold in patients with LDH levels **>**2 × ULN compared those with levels **<**1 × ULN, and 4.5-fold in patients with LDH levels **>**2 × ULN [8], suggesting that the higher the LDH level, the higher the risk of death. Furthermore, in Suh SY’ report, serum LDH levels increased in the terminal phase and rose significantly to 630.40 ± 417.32 IU/L (213–1047 IU/L) at 1 week before death [15], thus, LDH greater than 1000 U/L was a strong marker of terminal stage. Additionally, Pui CH divided 330 children with acute lymphoblastic leukemia into three groups defined by LDH levels: <300, 300 to 1000, and >1000 U/L. Patients with the highest LDH levels (>1000 U/L) were most likely to fail treatment and had the shortest time to failure [26]. Taken together, we established 1000 IU/L as the cut off value of LDH.

This study had several limitations. The enrolled patients were restricted to one local hospital, and the sample was relatively small to justify the effect of multiple clinical features on survival. The prognostic value of LDH level should be evaluated in a larger, multicenter setting. Our study, however, was of importance for its prediction of remaining life expectancy for patients with serum LDH level greater than 4 × ULN. And decrease in LDH level was significantly related to survival adjusted with other clinical variables. The prognostic value of LDH level should be evaluated in a larger, multicenter design.
